# Qili Qiangxin capsules for chronic heart failure: A GRADE-assessed clinical evidence and preclinical mechanism

**DOI:** 10.3389/fcvm.2022.1090616

**Published:** 2023-01-11

**Authors:** Xiaoxiao Xing, Jianbo Guo, Juefei Mo, Huashan Li, Hui Zhang, Baoyi Shao, Yifan Wang, Haidi Li, Jianan Wang, Cheuk Lung Leung, Yun Jiang, Weixian Yin, Haiyong Chen, Qingyong He

**Affiliations:** ^1^Guang’anmen Hospital, China Academy of Chinese Medical Sciences, Beijing, China; ^2^School of Chinese Medicine, LKS Faculty of Medicine, The University of Hong Kong, Hong Kong, Hong Kong SAR, China; ^3^Inflammation and Immune Mediated Diseases Laboratory of Anhui, School of Pharmacy, Anhui Institute of Innovative Drugs, Anhui Medical University, Hefei, China; ^4^Department of Chinese Medicine, The University of Hong Kong-Shenzhen Hospital, Shenzhen, China

**Keywords:** chronic heart failure, Qili Qiangxin, systematic review, myocardial apoptosis, adjuvant efficacy

## Abstract

**Introduction:**

Chronic heart failure (CHF) has become an increasing concern with the aging of the population. This study aims to evaluate the effectiveness and safety of Qili Qiangxin capsules (QLQX) for CHF.

**Methods:**

A systematic review and meta-analysis on clinical studies was conducted. The mechanisms of preclinical studies were summarized.

**Results:**

We searched six electronic databases by 20 July 2022, and finally, 7 preclinical experiments (PEs) and 24 randomized controlled trials were included. The risk of bias was accessed by the SYRCLE and RoB 2.0 tool, respectively. PEs indicated that QLQX suppresses myocardial apoptosis, inhibits renin-angiotensin-aldosterone system activation, improves water retention, and enhances cardiocyte remodeling. In clinical studies, compared with routine treatment, QLQX could improve the indicators: clinical efficacy rate (RR = 1.16, 95% CI [1.12, 1.22], GRADE: moderate), left ventricular end-diastolic dimension (SMD = −1.04, 95% CI [−1.39, −0.70], GRADE: low), left ventricular ejection fraction (SMD = 1.20, 95% CI [0.97, 1.43], GRADE: moderate), 6-minute walk distance (SMD = 1.55, 95% CI [0.89, 2.21], GRADE: low), brain natriuretic peptide (SMD = −0.78, 95% CI [−1.06, −0.51], GRADE: low), N-terminal pro-brain natriuretic peptide (SMD = −2.15, 95% CI [−3.60, −0.71], GRADE: low), and adverse events (RR = 0.46, 95% CI [0.25, 0.87], GRADE: low).

**Discussion:**

In summary, QLQX exerts a potential mechanism of utility on myocardial apoptosis and cardiac function and has noteworthy clinical adjuvant efficacy and safety in patients with CHF.

**Systematic review registration:**

https://www.crd.york.ac.uk/prospero/.

## 1. Introduction

Chronic heart failure (CHF) is a syndrome of venous blood stasis and arterial blood supply insufficiency due to impaired cardiac function and failure of pumping ventricular blood completely (1). The incidence of CHF has been increasing yearly with the aging of the population, and CHF is the end stage of various cardiac diseases. Routine treatment (RT) of CHF includes the use of diuretics to reduce cardiac load, angiotensin-converting enzyme inhibitors/angiotensin II receptor antagonists to reverse ventricular remodeling, β-blockers to inhibit sympathetic excitation, and digitalis drugs to inhibit Na+/K+-ATPase (1, 2). However, the contraindications of RT limit its use in CHF patients and result in a high risk of death or recurrence (3).

Oxidative stress and myocardial apoptosis are associated with the development and progression of CHF, particularly, the induction of HO-1 in CHF has been validated as an important cardioprotective adaptation against pathological left ventricular remodeling (4–6). QLQX is a Chinese patent medicine and has been commonly used for the treatment of CHF with wide acceptance in Chinese patients (7–9). It consists of Astragali Radix, Ginseng Radix et Rhizoma, Aconiti Lateralis Radix Preparata, Salvia Miltiorrhiza Radix et Rhizoma, Descurainiae Semen Lepidii Semen, Alismatis Rhizoma, Polygonati Odorati Rhizoma, Cinnamomi Ramulus, Carthami Flos, Periplocae Cortex, and Citri Reticulatae Pericarpium in the Chinese pharmacopeia. Notable that QLQX could exert cardioprotective effects by inhibiting the ROS/AMPK/mTOR signaling pathway to attenuate apoptosis and autophagic cell death (10). It has also been included in the Guidelines for Diagnosis and Treatment of Heart Failure in China, as a clinical recommendation for the treatment of CHF. Previous studies (11–13) have shown the promising effect of QLQX on CHF. This study aimed to explore the efficacy of QLQX for CHF and its molecular mechanisms through both preclinical and clinical aspects.

## 2. Materials and methods

### 2.1. Formulations and chemical

The inclusion of intervention in the study, QLQX, is based on the Chinese New Drug Certificate (National medicine permission number: Z20040141), and the patent “A pharmaceutical composition and preparation method for the treatment of chronic heart failure” (patent number: ZL02146573.8). The quality control, prescription composition and chemical composition involved in the included studies are shown in Supplementary Table 1.

### 2.2. Search strategy

This study was pre-registered (PROSPERO, No. CRD42021248464) and conducted following the PRISMA statement (14). The checklist was shown in Supplementary Table 2. The literature search was conducted in the databases of PubMed, Cochrane, Embase, Chinese National Knowledge Infrastructure (CNKI), China Science and Technology Journal (VIP), and Wanfang. The retrieval time was from the database establishment to 20 July 2021. MeSH terms combined with free search words were used for the literature search. The search strategy of the PubMed database was shown in Supplementary Table 3.

### 2.3. Eligible criteria

Inclusion criteria met the following requirements: (1) study type: preclinical experiments (PEs) or randomized controlled trials (RCTs); (2) cellular or animal models preclinically compatible with CHF, and patients clinically diagnosed with CHF; (3) QLQX alone or combined with RT as the intervention, and in preclinical experiments, the mechanism of QLQX was explored; (4) control group received RT or placebo; (5) there was no restriction on the outcome indicators of PEs, and the outcome indicators of RCTs were included as follows: clinical efficacy rate, cardiac function, 6-min walk distance (6-MWD), brain natriuretic peptide (BNP), N-terminal pro-brain natriuretic peptide (NT-proBNP), and adverse events. The exclusion criteria were as follows: (1) studies that included non-CHF patients; (2) repetitive studies, review, protocol, comment, case report, etc.; (3) PE or RCT included other Chinese medicine or related interventions in addition to QLQX; (4) data of studies was not available even though contacted with original authors.

### 2.4. Literature quality assessment

According to the Systematic Review Centre for Laboratory animal Experimentation (SYRCLE) (15), a risk of bias tool, PEs were assessed according to the following items: (1) sequence generation; (2) baseline characteristics; (3) allocation concealment; (4) random housing; (5) blinding of performance; (6) random outcome assessment; (7) blinding of detection; (8) incomplete outcome data; (9) selective outcome reporting; (10) other sources of bias. According to the Cochrane criteria, we assessed the quality of the included RCTs using the Risk of Bias 2.0 (RoB 2.0) (16) in six aspects: (1) bias arising from the randomization process; (2) bias due to deviations from intended intervention; (3) bias due to missing outcome data; (4) bias in measurement of the outcome; (5) bias in selection of the reported result; (6) overall. Under the supervision and coordination of a researcher (QH), two researchers (BS and YW) assessed the risk of the included PEs, and another two researchers (WY and YJ) assessed the risk of the included RCTs according to “low risk,” “high risk,” or “some concerns,” respectively.

### 2.5. Data extraction and analyses

The following data was extracted: (1) basic information of the included PEs (the first author, publication year, animal species, sex, number of animals, weight, intervention, experiment duration) and RCTs (the first author, publication year, sample size, age information of the patients, intervention, trial duration); (2) all outcome measures of experiments; (3) outcome measures including clinical efficacy rate, cardiac function, 6-MWD, BNP, NT-proBNP, and adverse events. Under the supervision of a researcher (HC), two researchers (HSL and JW) extracted the data of the PEs, and the other two researchers (CL and HZ) extracted the data of the RCTs.

The Stata 17.0 software (Stata Corp., College Station, TX, USA) was applied to statistical analysis: (1) random-effect model was employed, as different RT medicine or dosage may affect the therapeutic effect; (2) Cohen’s d and 95% CI were used for continuous variables; (3) relative risk (Relative Risk) and 95% CI were used for categorical variables; (4) A *p*-value < 0.05 indicated statistically significant; (5) Q statistics and *I*^2^ were used to evaluate the heterogeneity of each pooled analysis, and the *p*-value in Q statistics < 0.05 or *I*^2^ > 50% meant high heterogeneity; (6) the sensitivity analysis was carried out when any meta-analysis presented high heterogeneity; (7) as for the difference of interventions or classification of outcome measures, subgroup analyses were inclined to carry on.

### 2.6. GRADE assessment

This study used GRADE (Grades of Recommendation, Assessment, Development and Evaluation) (17), a transparent and structured quality rating system, to grade each of the outcome indicators in meta-analysis with four levels of evidence: high, moderate, low, and very low. The accessment followed the GRADE handbook. The reasons for downgrading levels of evidence certainty in the included studies were shown below: high risk of bias in results (18), high level of heterogeneity (19), low generalizability of the study (20), no statistical significance of effect size (21), and publication bias in the study (22).

## 3. Results

### 3.1. Eligible studies

**FIGURE 1:**
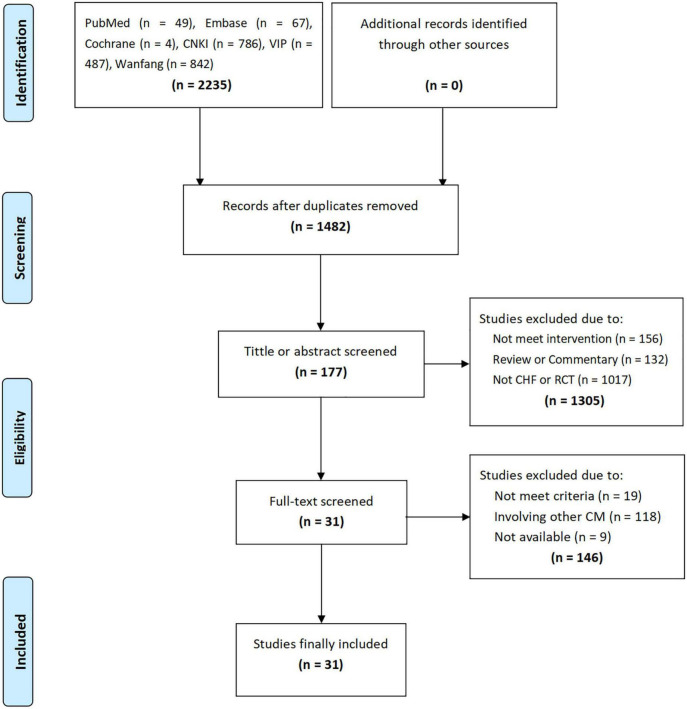
Flow chart.

A total of 2,235 studies were retrieved through the initial search, and 1,482 studies were obtained after removing duplicate studies. By screening the titles or abstracts, 177 studies were obtained, followed by the full-text accessment, and 7 PEs and 24 RCTs were included. The flow chart of the study screening is shown in Figure 1.

### 3.2. Literature characteristics

Seven PEs (23–29) and 24 RCTs (30–53) included 294 rats and 2,731 patients respectively. PE studies range from 4 to 8 weeks including 21–60 animals in each study. The clinical trial duration ranges from 2 to 24 weeks with patients from 64 to 491 each. In the PEs, all experiments were pharmacodynamic studies investing the biochemical effects of QLQX in animal CHF models. In the included RCTs, 1 trial (33) used the placebo in the control group. The characteristics of included studies are shown in Tables 1, 2.

**TABLE 1:** Basic information of the included preclinical experiments.

References	Preparation of the animal model	Animal species	Gender	Number of animals	Weight	Intervention	Experimental duration	Experimental type	Relevant targets
Lin (23)	Ligation operation	SD rats	Male	50	200–250 g	QLQX	4 weeks	Pharmacodynamic	Caspase-3
Xu (24)	Abdominal aorta coarctation	SD rats	Male plus female	60	200–240 g	QLQX	8 weeks	Pharmacodynamic	Fas, FasL, Bax, Bcl-2
Yu (25)	Ligation operation	Wistar rats	Male	42	230–250 g	QLQX	4 weeks	Pharmacodynamic	P2X
Qian (26)	Intraperitoneal injection of azithromycin in rats	Wistar rats	NA	21	200 ± 20 g	QLQX	6 weeks	Pharmacodynamic	Superoxide dismutase, malondialdehyde
Cui (27)	Ligation operation	SD rats	Male	40	200–250 g	QLQX	4 weeks	Pharmacodynamic	Aquaporin-2 expression and phosphorylation at serine 256
Li (28)	Ligation operation	Wistar rats	Male	21	250–280 g	QLQX	4 weeks	Pharmacodynamic	Superoxide dismutase
Liu (29)	Intraperitoneal injection of azithromycin in rats	Wistar rats	Male	60	245 ± 5 g	QLQX	4 weeks	Pharmacodynamic	CC chemokine receptor 4, G protein, Ca^2+^

SD, sprague dawley; QLQX, Qili Qiangxin capsule.

**TABLE 2:** Basic information of the included randomized controlled trials.

References	Sample size (T/C)	Mean age (years)	Diagnostic criteria	Intervention	Comparison	Duration of treatment	Outcomes
Ding (30)	64 (33/31)	T: 65.30 ± 11.70 C: 66.70 ± 13.10	Conform to I	QLQX plus RT	RT	2 weeks	⑦
Du (31)	106 (56/50)	T: 68.70 ± 4.70 C: 69.20 ± 4.50	Conform to I	QLQX plus RT	RT	4 weeks	④
Huang (32)	73 (37/36)	T: 63.18 ± 8.15 C: 61.27 ± 8.09	Conform to I	QLQX plus RT	RT	4 weeks	⑦
Li (33)	491 (244/247)	T: 56.98 ± 11.59 C: 57.53 ± 11.05	Conform to I	QLQX plus RT	RT plus placebo	12 weeks	① ② ③ ⑥
Guo (34)	80 (42/38)	T: 59.20 ± 4.30 C: 60.10 ± 6.30	Conform to I	QLQX plus RT	RT	8 weeks	① ② ⑤
Kong (35)	78 (39/39)	55.60 ± 12.70	Conform to I	QLQX plus RT	RT	12 weeks	① ② ③
Liu (36)	80 (40/40)	T: 56.23 ± 2.50 C: 54.54 ± 3.70	Conform to I	QLQX plus RT	RT	4 weeks	①
Gao (37)	88 (44/44)	T: 61.08 ± 8.41 C: 60.46 ± 8.32	Conform to I	QLQX plus RT	RT	12 weeks	① ② ③ ⑤
Wang (38)	90 (45/45)	T: 61.40 ± 8.20 C: 62.30 ± 7.70	Conform to I	QLQX plus RT	RT	8 weeks	① ④
Li (39)	86 (43/43)	T: 67.80 ± 1.50 C: 68.00 ± 1.50	Conform to I	QLQX plus RT	RT	4 weeks	① ④
Liu (40)	80 (40/40)	T:62.10 ± 9.20 C:61.40 ± 9.30	Conform to I	QLQX plus RT	RT	24 weeks	①
Liu (41)	78 (39/39)	T:62.01 ± 5.62 C:61.68 ± 5.79	Conform to I	QLQX plus RT	RT	8 weeks	① ② ⑥
Dong (42)	120 (60/60)	T: 68.90 ± 4.56 C:71.30 ± 5.62	Conform to I	QLQX plus RT	RT	12 weeks	② ⑤
Han (43)	76 (38/38)	T:58.97 ± 5.33 C:57.82 ± 5.41	Conform to I	QLQX plus RT	RT	12 weeks	① ④
Li (44)	70 (35/35)	T:38.50 ± 4.10 C:35.20 ± 3.90	Conform to I	QLQX plus RT	RT	8 weeks	① ④ ⑥
Lu (45)	106 (53/53)	T:62.19 ± 9.82 C:62.54 ± 9.11	Conform to I	QLQX plus RT	RT	12 weeks	① ② ③ ④ ⑥
Li (46)	107 (54/53)	T: 60.17 ± 2.32 C:60.22 ± 2.53	Conform to I	QLQX plus RT	RT	12 weeks	① ② ③ ⑤
Liu (47)	90 (45/45)	T: 74.65 ± 5.57 C: 73.45 ± 4.64	Conform to I	QLQX plus RT	RT	4 weeks	② ④ ⑥
Liu (48)	128 (64/64)	T:56.42 ± 8.57 C:57.46 ± 7.56	Conform to I	QLQX plus RT	RT	12 weeks	⑤ ⑥
Nie (49)	82 (41/41)	T:58.72 ± 4.67 C:58.67 ± 4.72	Conform to I	QLQX plus RT	RT	12 weeks	① ② ④ ⑥
Sun (50)	66 (33/33)	T:60.58 ± 6.33 C:61.44 ± 6.25	Conform to I	QLQX plus RT	RT	8 weeks	① ② ③
Yang (51)	86 (43/43)	T: 62.64 ± 1.23 C:62.58 ± 1.17	Conform to I	QLQX plus RT	RT	8 weeks	① ⑤ ⑥
Wang (52)	92 (46/46)	T:69.89 ± 5.61 C:70.40 ± 5.38	Conform to I	QLQX plus RT	RT	8 weeks	① ② ⑤ ⑥
Yao (53)	108 (54/54)	T: 60.34 ± 8.94 C:59.83 ± 7.58	Conform to I	QLQX plus RT	RT	4 weeks	① ② ③ ⑤ ⑥

T, treatment group; C, control group; QLQX, Qili Qiangxin capsule; RT, routine treatment (including ACEI, angiotensin-converting enzyme inhibitor, beta-blocker; MRA, mineralocorticoid receptor; ARB, angiotensin receptor blocker, diuretics, cardiac agents, vasodilating agent); ①: Clinical efficacy rate; ②: Cardiac function; ③: 6 min walk distance (6-MWD); ④: BNP; ⑤: NT-proBNP; ⑥:Adverse effect; ⑦: Na^+^; I: 2021ESC Guidelines for the diagnosis and treatment of acute and chronic heart failure: The task force for the diagnosis and treatment of acute and chronic heart failure of the european society of cardiology (ESC) Developed with the special contribution of the heart failure association (HFA) of the ESC.

### 3.3. Risk of bias

Seven PEs (23–29) were assessed by the SYRCLE risk of bias tool (Supplementary Table 4). All PEs were assessed as the “some concerns” in the item of “random housing” in all PEs due to lack of reporting. Three PEs (27–29) existed a “some concerns” risk in the “sequence generation” section due to lack of group method reporting, and other two PEs (25, 26) without baseline information were assessed as the “some concerns.” A total of 24 RCTs (30–53) were assessed by RoB 2.0 risk of bias tool (Supplementary Table 5). All RCTs were assessed as the “low” risks in the item of “bias due to deviations from intended intervention” and “bias arising from the randomization process.” From the item of “bias in measurement of the outcome” or “bias in selection of the reported result,” three RCTs (38, 42, 49) were assessed as “high” risks because of incomplete data and results.

## 4. Preclinical experiments

**FIGURE 2:**
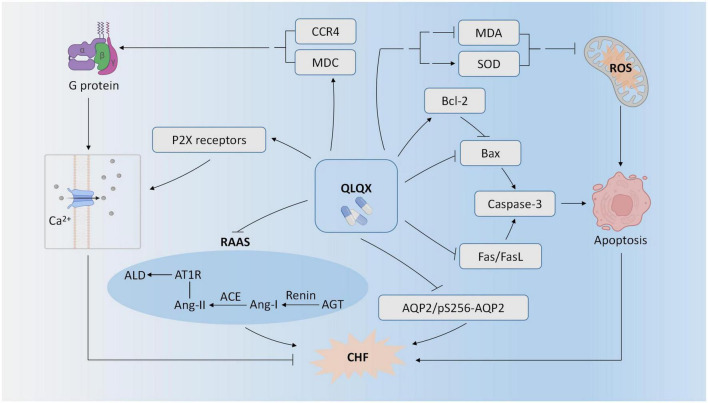
Potential mechanism of QLQX for CHF.

Seven PEs (23–29) studied the effect of QLQX for CHF in the animal models. As shown in Figure 2, one experimental study showed that QLQX improved cardiac function in the CHF rat model of the left anterior descending coronary artery ligation by suppressing caspase-3 mediated myocardiocyte apoptosis (23). Consistently, QLQX attenuated apoptosis in myocardiocytes of CHF rats by inhibiting Fas, FasL, and Bax and upregulating Bcl-2 (24). Two experimental studies indicated that QLQX reduced levels of oxidative stress in CHF rats as demonstrated by the downregulation of malondialdehyde (MDA) and upregulation of superoxide dismutase (SOD) in serum (26, 28). QLQX also improved cardiac function by inhibiting activation of cardiac RAAS, e.g., upregulation of renin, angiotensin II, and aldosterone, angiotensin converting enzyme (ACE), and angiotensin type 1 receptor (AT1R) (28). QLQX elevated MDC protein content and expression, and the binding of MDC to CC chemokine receptor 4 (CCR4) could activate G protein and promote Ca^2+^ influx, finally enhancing myocardial contractile function (29). QLQX improved cardiac output by increasing P2X-mediated ventricular contractility in CHF rats (25). QLQX also reduced water reabsorption by suppressing aquaporin-2 expression and phosphorylation at serine 256 (pS256-AQP2) (27).

## 5. Clinical trials

### 5.1. Clinical efficacy rate

**FIGURE 3:**
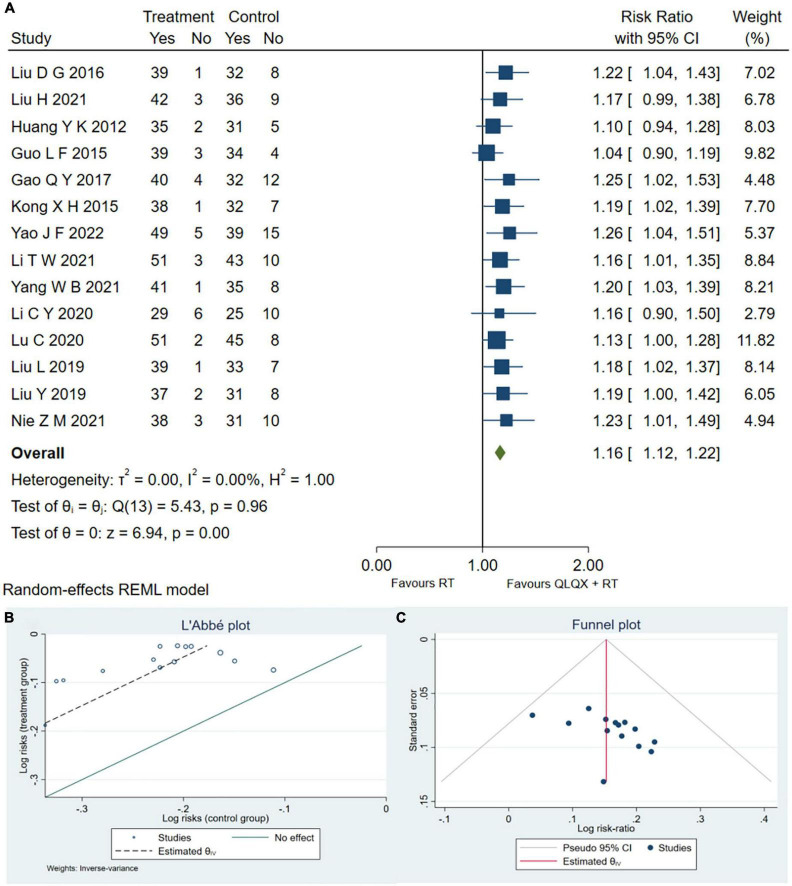
**(A)** Forest plot of the clinical efficacy rate; **(B)** L’Abbe plot; **(C)** Funnel plot.

Fourteen RCTs (32, 34–37, 40, 41, 44–47, 49, 51, 53) reported the clinical efficacy rate. As shown in Figure 3A, QLQX plus RT significantly increased clinical efficacy rate [RR = 1.16, 95% CI (1.12, 1.22), *p* < 0.05, GRADE: moderate, as shown in Table 3], with no heterogeneity [*Q* (13) = 5.43, *P* = 0.00, *I*^2^ = 0]. Egger test results showed that there was no published bias (β_1_ = 2.02, SE of β_1_ = 1.636, *z* = 1.23, *P* = 0.2180 > 0.05). The L’Abbe and funnel plots are respectively presented in Figures 3B, C.

**TABLE 3:** GRADE summary of comparing QLQX plus RT group with RT groups.

Outcomes	Anticipated absolute effects (95% CI)		Relative effect (95% CI)	No of participants (studies)	Certainty of the evidence (GRADE)
	Assumed risk: non-acupuncture	Corresponding risk: acupuncture			
Clinical efficacy rate	798 per 1000	846 per 1000 (830–854)	RR 1.06 (1.04–1.07)	1205 (14 RCTs)	⊕⊕⊕○ MODERATE
6-MWD	The mean 6-MWD in the control groups was 92.82	The mean weekly defecation in the intervention groups was 1.55-fold higher (0.89–2.21-fold higher)	–	573 (6 RCTs)	⊕⊕○○ LOW
BNP	The mean BNP in the control groups was 635.09	The mean BNP in the intervention groups was −0.78-fold higher (−1.06 to −0.51-fold higher)	–	554 (8 RCTs)	⊕⊕○○ LOW
NT-proBNP	The mean NT-proBNP in the control groups was 2662.80	The mean NT-proBNP in the intervention groups was −2.15-fold higher (−3.60 to −0.71-fold higher)	–	689 (6 RCTs)	⊕⊕○○ LOW
LVEDD	The mean LVEDD in the control groups was 4.72	The mean LVEDD in the intervention groups was −1.04-fold higher (−1.39 to −0.70-fold higher)		981 (11 RCTs)	⊕⊕○○ LOW
LVEF	The mean LVEF in the control groups was 6.73	The mean LVEF in the intervention groups was 1.20-fold higher (0.97 to 1.43-fold higher)		1183 (14 RCTs)	⊕⊕⊕○ MODERATE
LVESD	The mean LVESD in the control groups was 6.87	The mean LVESD in the intervention groups was 0.10-fold higher (−0.35 to 0.56-fold higher)		254 (3 RCTs)	⊕⊕○○ LOW
Adverse events	223 per 1000	102 per 1000 (56–194)	RR 0.46 (0.25–0.87)	619 (7 RCTs)	⊕⊕○○ LOW

RCTs, randomized controlled trials; LOW (low certainty): Our confidence in the effect estimate is limited: The true effect may be substantially different from the estimate of the effect; MODERATE (moderate certainty): We are moderately confident in the effect estimate: The true effect is likely to be close to the estimate of the effect, but there is a possibility that it is substantially different. 6-MWD, 6-min walk distance.

### 5.2. Cardiac function

**FIGURE 4:**
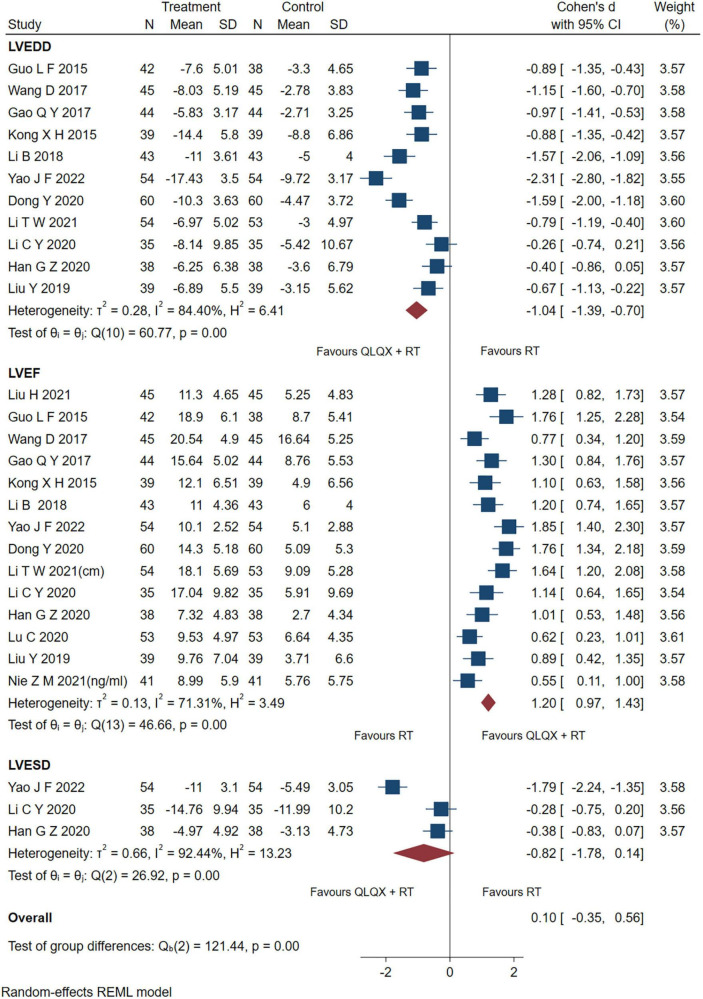
Forest plot of the cardiac function.

Fourteen studies reporting the results of LVEF, 7 studies reporting LVEDD and three studies (43, 44, 53) reporting LVESD were pooled and analyzed. As shown in Figure 4, the intervention group significantly improved the indicators of LVEF [SMD = 1.20, 95% CI (0.97, 1.43), *p* < 0.05; *Q* (13) = 46.66, *p* = 0.00, *I*^2^ = 71.31%, GRADE: moderate], and LVEDD [SMD = −1.04, 95% CI (−1.39, −0.70), *p* < 0.05; *Q* (10) = 60.77, *p* = 0.00, *I*^2^ = 84.40%, GRADE: low] compared to the control group. However, there was no significant difference between QLQX plus RT and RT on decreasing LVESD in patients with CHF [SMD = −0.82, 95% CI (−1.78, 0.14), *p* > 0.05; *Q* (2) = 26.92, *p* = 0.00, *I*^2^ = 92.44%, GRADE: low]. Heterogeneity analysis indicated that differences in drug dosage and sample size might cause high heterogeneity.

### 5.3. 6-min walk distance

**FIGURE 5:**
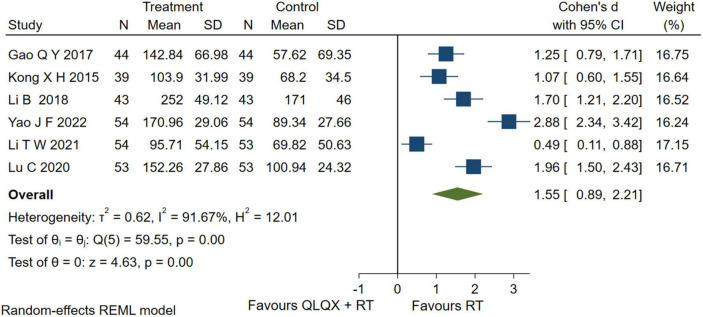
Forest plot of 6-min walk distance.

Six studies (35, 37, 39, 45, 46, 53) reported the 6-MWD. The forest plot in Figure 5 showed that intervention group improved the 6-MWD significantly [SMD = 1.55, 95% CI (0.89, 2.21), *p* < 0.05, GRADE: low]; but there was a higher heterogeneity [*Q* (5) = 59.55, *p* = 0.00, *I*^2^ = 91.67%]. The heterogeneity analysis indicated that different durations led to high heterogeneity.

### 5.4. BNP and NT-proBNP

**FIGURE 6:**
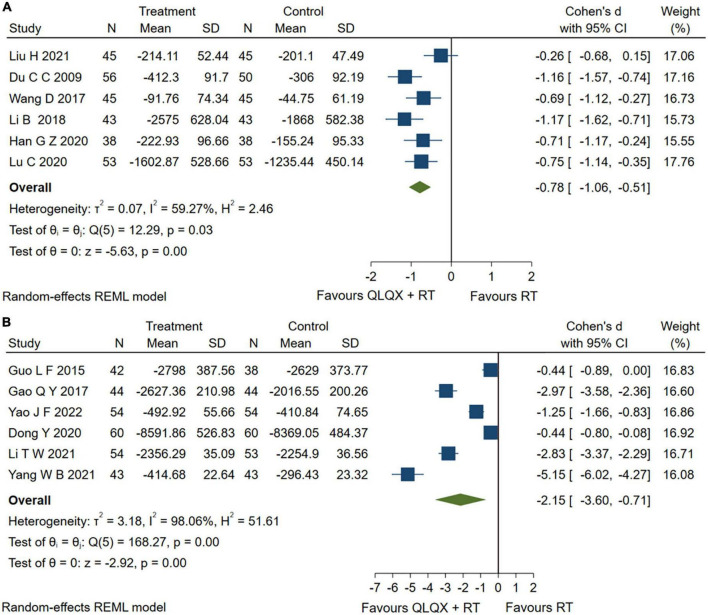
**(A)** Forest plots of brain natriuretic peptide; **(B)** N-terminal pro-brain natriuretic peptide.

Eight studies (31, 38, 39, 43–45, 47, 49) reported serum BNP levels. However, sensitivity analysis found that two studies (44, 49) caused a higher heterogeneity [*Q* (7) = 152.55, *p* = 0.01, *I*^2^ = 98.16%] and were excluded from the pooled analysis. The forest plot in Figure 6A reported that QLQX plus RT significantly reduced the level of serum BNP in patients with CHF [SMD = −0.78, 95% CI (−1.06, −0.51), *p* < 0.05, *Q* (5) = 12.29, *p* = 0.00, *I*^2^ = 59.27%, GRADE: low]. Six studies (34, 37, 42, 46, 51, 53) QLQX significantly decreased serum NT- proBNP levels [SMD = −2.15, 95% CI (−3.60, −0.71), *p* < 0.05, *Q* (5) = 168.27, *p* = 0.00, *I*^2^ = 98.06%, GRADE: low] as shown in Figure 6B.

### 5.5. Adverse events

**FIGURE 7:**
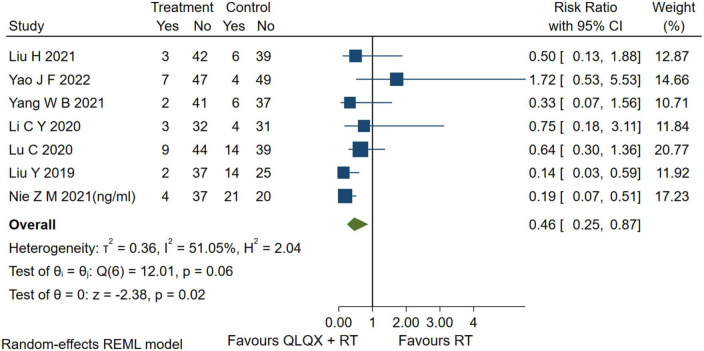
Forest plot of adverse events.

Seven studies (41, 44, 45, 47, 49, 51, 53) reported adverse events. The forest plot Figure 7 indicated that compared with the RT, QLQX treatment showed a decrease in adverse events [RR = 0.46, 95% CI (0.25, 0.87), *p* < 0.05, *Q* (6) = 12.01, *p* = 0.02, *I*^2^ = 51.05%, GRADE: low].

## 6. Discussion

From the clinical aspect of traditional Chinese medicine, QLQX could be used for the type of heart-Yang deficiency caused by Qi inadequacy and blood stasis in CHF (33). This study explored the possible molecular mechanism and clinical efficacy of QLQX in the treatment of CHF. In the clinical section, our pooled analysis showed that QLQX presented significant efficacy in clinical efficacy rate, LVEF, LVEDD, 6-MWD, BNP, NT-proBNP, and adverse events. Previous evidence (54) has shown that tailor-made medications by NT-proBNP guided approach reduce all-cause mortality of CHF by 20% compared with conventional treatments. Our findings show that QLQX has a larger reductive effect on NT-proBNP, suggesting a good clinical effect on CHF. The GRADE-assessed evidence added to this study demonstrates that most of the pooled analyses remain low-level evidence recommendations but show a moderate level of evidence in clinical efficacy rate and improved LVEF indicator due to the sufficient number of studies and patients.

Based on our preclinical findings and previous studies, PI3K/AKT may be a noteworthy signaling pathway regulated by QLQX during the treatment of CHF. QLQX could activate this signaling pathway, up-regulating GSK3β, increasing Bcl-2 and decreasing Bax (55, 56). The pathway could also activate enzymatic antioxidant systems, including SOD, catalase, and heme oxidase-1, which prevents the excessive production of ROS, and reduce the level of MDA. And SODs could catalytic O^2–^ to H_2_O_2_, then catalase or glutathione peroxidase in the cytoplasm and mitochondria converts H_2_O_2_ to H_2_O and O_2_ (56, 57). Besides, it has been proved that the accumulation of ROS could induce apoptosis and excessive autophagy, while QLQX could inhibit it by down-regulating AMP-activated protein kinase phosphorylation, and up-regulating mechanistic target of rapamycin (mTOR) phosphorylation (10). Another study (58) indicates that the cardioprotective effect of QLQX is partially mediated through PI3K/AKT-dependent vascular endothelial-derived growth factor (VEGF) expression, which could prevent apoptosis and regulate cell survival. According to previous studies (59, 60), although the role of p53 gene in the heart is well established, it is not clear how p53 is regulated in CHF. In normal cells, p53 expression is kept at low levels by the E3 ubiquitin ligase mouse double minute 2 homolog (Mdm2), which targets p53 for proteasomal degradation. In response to acute stress, Mdm2 is inactivated and increased p53 levels block cell division and induce apoptosis (61). Therefore, we hypothesize that QLQX could downregulate p53 to curb the negative feedback loop of p53 activity, but further validation is needed.

There are some limitations in this study. First, even though one high-quality RCT (33) is included, the overall quality of the included RCTs is still not enough to provide a high certainty of evidence. Second, mechanisms of QLQX protecting against CHF are not fully explored. In the study, low to moderate levels of evidence indicated that QLQX attenuates CHF. In the future, more well-designed RCTs should be conducted to improve the reliability of evidence. Preclinical studies should delve into the signaling pathways associated with ventricular remodeling to explore the mechanisms by which QLQX exerts its effectiveness as well as determine the active compounds in QLQX.

## 7. Conclusion

QLQX has adjuvant efficacy and safety in patients with CHF and improves clinical efficacy rates, LVEF at moderate levels of evidence. QLQX could attenuate CHF by inhibiting apoptosis, suppressing RAAS activation, improving water retention, and enhancing cardiocyte remodeling.

## Data availability statement

The original contributions presented in this study are included in the article/Supplementary material, further inquiries can be directed to the corresponding authors.

## Author contributions

QH and HC conceived and designed the study. HC revised the manuscript. JM and HDL supervised the study and provided some comments. XX and JG performed the data analysis and wrote the manuscript. BS, YW, WY, and YJ assessed the literature. HSL, JW, CL, and HZ extracted the data. All authors contributed to the article and approved the submitted version.
